# Epidemiology of Major Depressive Disorder in Iran: a Systematic Review and Meta-Analysis

**Published:** 2010

**Authors:** Behnam Sadeghirad, Ali-Akbar Haghdoost, Masoumeh Amin-Esmaeili, Esmaeil Shahsavand Ananloo, Padideh Ghaeli, Afarin Rahimi-Movaghar, Elham Talebian, Ali Pourkhandani, Ahmad Ali Noorbala, Esmat Barooti

**Affiliations:** 1 Neuroscience Research Centre, Kerman University of Medical Sciences (KUMS), Kerman, Iran; 2 Department of Epidemiology and Biostatistics, School of Health, KUMS, Kerman, Iran; 3 Iranian Research Center for HIV/AIDS (IRCHA), Tehran University of Medical Sciences (TUMS), Tehran, Iran; 4 Psychiatric Research Center, Roozbeh Psychiatric Hospital, TUMS, Tehran, Iran; 5 Psychiatric Research Center, Roozbeh Psychiatric Hospital, TUMS, Tehran, Iran; 6 Medical Students Research Center, KUMS, Kerman, Iran; 7 Women Health Affairs Office, Ministry of Health and Medical Education, Tehran, Iran

**Keywords:** Depressive disorder, Mental health, Epidemiology, Prevalence, Review, Meta-analysis

## Abstract

**Objectives:**

There are a large number of primary researches on the prevalence of major depressive disorder (MDD) in Iran; however, their findings are varied considerably. A systematic review was performed in order to summarize the findings.

**Methods:**

Electronic and manual searches in international and Iranian journals were conducted to find relevant studies reporting MDD prevalence. To maximize the sensitivity of the search, the references of relevant papers were also explored. We explored the potential sources of heterogeneity such as diagnostic tools, gender and other characteristics using meta-regression model. The combined mean prevalence rates were calculated for genders, studies using each type of instruments and for each province using meta-analysis method.

**Results:**

From 44 articles included in the systematic review, 24 reported current prevalence and 20 reported lifetime prevalence of MDD. The overall estimation of current prevalence of MDD was 4.1% (95% CI: 3.1-5.1). Women were 1.95 (95% CI: 1.55-2.45) times more likely to have MDD. The current prevalence of MDD in urban inhabitants was not significantly different from rural inhabitants. The analysis identified the variations in diagnostic tools as an important source of heterogeneity.

**Conclusions:**

Although there is not adequate information on MDD prevalence in some areas of Iran, the overall current prevalence of MDD in the country is high and females are at the greater risk of disease.

## INTRODUCTION

Depression is the leading cause of disability^1^, and an important global public health issue. It was accounted for 4.4% of the total global disability adjusted life years (DALYs) in 2000^2^ and is projected as the second leading cause of burden of disease in 2020^3^ and 2030.^4^ In addition, depression was associated with an increased mortality risk of 1.8 (1.6-2.1) in a meta-analysis of 25 community surveys involving more than 100,000 participants.^5^ International comparisons among countries have shown considerable variations in the prevalence of major depressive disorder (MDD).^6^ According to the findings of World Mental Health (WMH) Survey Initiative, among low and middle income countries, life time prevalence of MDD was 7.2%, 14.6%, 3.5% and 3.3% in Iraq^7^, Ukraine^8^, China^9^ and Nigeria^10^, respectively. Among high-income countries, in Spain^11^, Japan^12^ and USA^13^ reported rate have been 10.5%, 6.7% and 16.2%, respectively. Twelve month prevalence of MDD has been reported to be 2% in China^14^, 2.9% in Japan^15^, 8.3% in Ukraine^8^, 5.7% in New Zealand^16^, 3.9% in Spain^11^, 3.7% in Mexico^17^ and 1% inNigeria.^10^ Iran is one of the widest countries in the Middle East region, with more than 70 million population from several ethnicities. Azari people are living mostly in north-west, Kurdish in west, Arabs in south and south-west, Fars in center, Turkmen in north-east, and Balouch in east part of the country. The differences in the cultures, lifestyles, and socioeconomic status might cause variations in the prevalence of depressive disorders. In addition, Iran has experienced profound socioeconomic changes over the past three decades.^18^ Such evolutions during the past decades might have influenced health, and accordingly the prevalence of common mood disorders. So far, two national surveys on the prevalence of mental disorders have been conducted in Iran.^19^,^20^ The results of the surveys were considerably different. A systematic review was performed on the prevalence of mental disorders in the year 2006 and discussed the differences in various studies conducted in the country.^21^ The review showed the necessity for using all evidences from the country to conclude on the prevalence of the mental disorders. A number of small- to intermediate-scale epidemiologic studies as well as a national survey have reported the prevalence of MDD around the country. This study aimed to review the findings of all available studies, systematically and to estimate the overall prevalence of MDD and to examine the potential sources of heterogeneity in Iranian population.

## METHODS

### Search strategy

Adhering to the Quality of Reporting of Meta-analyses (QUOROM) guideline21 and compatible with guidelines presented by Khan et al.^22^, we searched Medline through PubMed www.ncbi.nlm.nih.gov), ISI Web of Science www.isiknowledge.com), Scopus www.scopus.com), PsycNET (psycnet.apa.org), EMBASE www.embase.com/search), Science Direct www.sciencedirect.com),and Scholar Google. Besides, we searched Iranian databanks including: Iran Psych, www.iranpsych.tums.ac.ir), Iranmedex www.iranmedex.com), and IranDoc www.irandoc.ac.ir). IranPsych databank provides soft or hard copies of articles published in peer-reviewed journals and postgraduate theses, as well as abstracts of scientific articles presented in scientific congresses and reports of research projects related to mental health. Iranmedex, which is a nonspecialized databank, provides research articles in medical sciences and encompasses articles published in scientific journals of Iran. Iranian Research Institute for Scientific Information and Documentation (IranDoc) contains abstracts of research projects, Iranian dissertations, scientific and technical articles in all fields of science. All available Iranian psychiatric journals were also hand searched; moreover, the reference lists of retrieved studies were screened for additional relevant studies. We did not impose any restrictions on time of study or language and publication status. Contacting authors to access missing data was also extensively performed. A search strategy that combines a highly sensitive filter for observational studies and subject-specific terms were used. For searching international databases, English transcription of Iran, large cities and the name of the universities were used^21^ and combined with the terms related to epidemiology and prevalence (epidemiolog* OR survey OR prevalence OR screening), and the terms related to mood disorders (Mood OR affective OR depress* OR dysthymi* OR cyclothymi* OR bipolar* OR mani* OR dysphori*). Iranian databanks were searched for related studies with the same strategy. The Persian keywords were equivalent to their English words and all probable combinations were considered. Considering the limitation in the use of a large number of keywords and the simultaneous use of the logical operators “and” and “or”, each group of keywords were separately searched in the databanks and duplicated documents were removed. All searching procedures were completed between Oct and Dec 2008.

### Selection criteria

Two reviewers reviewed all identified titles and abstracts, independently. The full texts of all articles considered relevant by either reviewer were then assessed. Studies were independently selected and were included in case they were original and provided estimates of lifetime or current prevalence of MDD in general population over 15 years. The studies were included if they had used a standardized interview or validated questionnaire to diagnose MDD. As the definition for current prevalence of MDD was different in the studies, we considered all definitions using a period of less than 12 months, as well as studies using the term of current, without any definition as “current prevalence of MDD”. Studies were excluded if they were not primary studies (such as reviews). We also excluded studies, which were not representative of adult general population, such as studies conducted on specific population subgroups for example, medically ill patients, women in menopausal period, pregnant or parturient women, refugees, university students, prisoners, institutionalized patients, and individuals in specific jobs. Studies that only relied on screening tools for determining depressive disorders, reported the prevalence of MDD in a small sample (less than 200 individuals), or in a non-random sample were also excluded. Discrepancies between reviewers were resolved by consensus.

### Quality assessment and data extraction

To assess the quality of included studies, a simple checklist based on Boyle study^25^ was used and studies were evaluated for main issues in descriptive studies such as the sampling method, and the validity of diagnostic tools. Two investigators independently extracted data, reconciling differences by consensus. Following information was extracted: publication and study date, study population and location, sample size, age of participants, used instrument, diagnosis based on DSM or ICD criteria, current and lifetime prevalence of MDD.

### Analytic strategies

The variance of current prevalence of MDD in each study was computed based on the binomial distribution formula. As significant heterogeneity was detected, we applied random effect model to estimate the combined mean prevalence of MDD. In addition, in order to minimize the random variation between point estimations of studies, we adjusted all findings of the studies using Bayesian analysis. In this adjustment, the overall point estimation based on the random effect model was used as prior prevalence. In next step, we used meta-regression to check the effect of gender, type of instrument used to assess depression, living location of participants (rural vs. urban area), publication and study date as possible sources of heterogeneity among the studies. We estimated tau-square (τ^2^), using restricted likelihood method, as the indicator of heterogeneity. A significance level of <0.10 and τ^2^>50% was considered as heterogeneity. In the case of significant heterogeneity, the random effect model was used and the relevant factors were explored in subgroup analysis where data was available. The analyses were performed using STATA software, version 8 (College station, TX: Stata corporation, 2003) and Review Manager (RevMan) version 5.0 (Copenhagen: the Nordic Cochrane center, the Cochrane collaboration, 2008).

## RESULTS

### Description of studies

**Figure 1:**
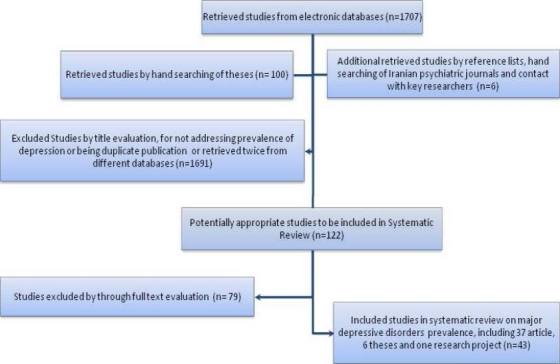
The flow chart of study selection

The number of identified studies using different sources and the stages of evaluation and exclusion are presented in Figure 1.

Excluded ones were the studies that had not used diagnostic tools to assess the prevalence of MDD (33 studies), the samples were not representative of general population (17 studies) and specific data on MDD prevalence could not be obtained despite contacting the authors (6 studies). Besides, the other articles were excluded for the following reasons: double publication (3 studies), reviews (3 studies) and sample size of less than 200 (4 studies). Others were excluded for not fulfilling the other inclusion criteria. Forty-three studies met the inclusion criteria for this review. Nineteen studies were part of one national household survey conducted in 2001 and reported results for different provinces. This survey was the only study that assessed the lifetime prevalence of MDD and used Schedule for Affective Disorders and Schizophrenia (SADS) based on DSM-IV criteria.^22^ Twenty-four studies reported the current prevalence of MDD. Almost all studies have been conducted in two phases, which used SCL-90, GHQ-28, Beck, SQR and Duck as screening tools and followed by a DSM-III or DSM-IV based clinical interview for confirmation. Only one study on the national level was performed in one phase using SADS as a diagnostic tool, which was based on DSM-IV criteria. The studies on current prevalence of MDD were conducted from 1992 to 2004. Nearby half of the studies had been conducted before 2000. The target population in 12 studies lived in urban areas, while seven studies reported the prevalence of MDD in rural populations. The rest of included studies (5/24) had recruited samples from both urban and rural areas. The characteristics of the studies on current prevalence of MDD are summarized in Table 1.

**Table 1:** Study characteristics and results of included studies on current prevalence of major depression in Iran

First author [ref]	Study date	Province (city/village)	Age group	Study population	Instrument/questionnaire	Sample size	Prevalence
Modabernia MJ 23	2003-2004	Guilan (Rasht)	18-70 y/o	General population, urban/rural with family files registered in health center	Beck & DSM IV based clinical interview	3578	1.2
Sadeghi KH 24	2003	Kermanshah (Kermanshah)	≥ 15 y/o	Urban residents	SRQ & DSM IV based clinical interview checklist	2400	4.21
Hassanshahi MM 25	2002	Fars (Arsanjan)	≥ 15 y/o	General population (urban & rural) with family files registered in health center	SCL-90 R, DSM IV based clinical interview	650	7.23
Hosseinifard SM 26	2001-2002	Kerman (Rafsanjan)	15-18 y/o	High school students	SCL 90 & DSM IV based clinical interview checklist	830	2.4
Omidi A 27	2001	Isfahan (Na tanz)	≥ 15 y/o	Urban households with family files registered in health center	GHQ-28, DSM IV clinical interview checklist	650	3.4
Kaviani H 28	2000	Tehran (Tehran)	20-65 y/o	Urban residents	Beck & DSM IV clinical interview checklist	1053	6.8
Shamsalizadeh N 29	2000	Tehran (Valian village)	≥ 15 y/o	Rural households	GHQ 28 & DSM IV clinical interview checklist	629	11.3
Nazari H 30	2000	Tehran (Tehran)	20-64 y/o	Residents of the city	Beck & DSM IV based clinical interview	1191	6.7
Fakhari A 31	2000	East-Azarbayjan (North west of Tabriz)	> 16 y/o	General population, urban residents with > 30 Duke questionnaire score or ≥ 5 in depression subscale	Duke & DSM IV based clinical interview	2270	2.7
Mohammadi A 32	2000	Isfahan (Shahin-shahr)	15 -18 y/o	High school students	SCL 90R & DSM IV based clinical inter view	1105	0.72
Noorbala A 33	1999	Tehran (Tehran)	> 15 y/o	Urban house holds	GHQ-28, DSM IV clinical interview checklist	879	4.4
Hassanzadeh Gh 34	1998-1999	East-Azarbayjan (Oskou)	≥ 15 y/o	Urban residents	SCL 90 R & DSM IV based clinical inter view	252	1.2
Sadeghi K 35	1997	Kermanshah (Kermanshah)	≥ 15 y/o	Urban households with family file registered in health center	SQR, DSM IV based clinical interview	501	1.6
Hosseini SR 36	1996	Tehran (Ta leghan)	15-25 y/o	High school students	GHQ 28 & DSM IV based structured inter view	200	5
Ghassemi GR 37	1996	Isfahan (Isfahan)	≥ 15 y/o	Adult urban households	SRQ & DSM IV based clinical interview checklist	3255	5.96
Yaghoubi N 38	1995	Guilan (Sou mehsara)	≥ 15 y/o	General population (urban & rural) with family files registered in health center	GHQ-28, DSM III based structured inter view	625	6.24
Attari A 39	1994	Isfahan (Sha hreza)	≥ 20 y/o	Urban residents	Beck & DSM III R based clinical interview	1269	13.6
Palahang H 40	1994	Isfahan (Kashan)	≥ 15 y/o	Urban house holds	GHQ-28, DSM III R clinical interview checklist	619	4.2
Khosravi Sh 41	1994	Charmahal Bakhtiary (Broujen)	≥ 15 y/o	Urban & rural households	SCL-90 R, DSM III R based interview, Davi dian clinical interview form	450	4.66
Bahadorkhan J 42	1993	Khorasan (Gonabad)	≥ 15 y/o	Rural residents with family files registered in health center	SCL 90 R & DSM III R based clinical interview	465	2.58
Javidi H 43	1993	Fars (Marvdasht)	≥ 15 y/o	Rural household with family files registered in health center	SCL 90 & DSM III R based clinical inter view	407	1
Kokabeh F 44	1993	East-Azarbayjan (Azar-shahr)	≥ 15 y/o	Rural households with family files registered in health center	SCL 90 & DSM III R based clinical interview	415	0.7
Bagheri SA 45	1992	Yazd (Meibod)	≥ 15 y/o	Rural people with family files registered in health center	SCL 90 R & DSM III R based clinical interview & Davidian Interview form	400	0.25
Mohammadi MR 46	2001	National	≥ 18 y/o	Urban & rural households	SADS/ DSM IV based structured interview	25180	1.8

### Lifetime prevalence of MDD

The only study that reported the lifetime prevalence for MDD was a national survey with a sample size of 25,180 conducted on the population above 18 years. The rate was reported to be 2.98% (95% CI: 2.8-3.2) and 3.10% (95% CI: 2.9-3.3) in two different published papers.^19^,^22^ The rate was from 0.3 percent to 5.59 percent in different provinces. Overall, the prevalence was higher in central as well as northwestern region. The lifetime prevalence of MDD was 4.38% (95% CI: 4.03- 4.76) in women and 1.59% (95% CI: 1.38-1.82) in men.^19^ The prevalence was significantly higher in urban areas (odds ratio=1.17, 95% CI: 1.01-1.37) than in rural areas.^22^ Due to the lack of any further published paper on the lifetime prevalence of MDD, we did not perform meta-analysis for this category.

### Current prevalence of MDD

**Figure 2:**
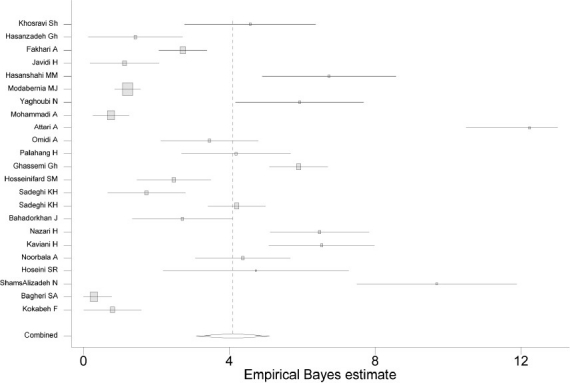
The forest plot of current prevalence of major depressive disorder in Iran

The total sample size for all of the 24 included studies on current prevalence of MDD was 49,273. One of the studies, the national household survey included 25,180 individuals. In the other studies, the maximum and minimum sample sizes of the studies were 3,578, and 200 in Guilan province and Taleghan district in Tehran province, respectively. Table 1 shows the characteristics and results of included studies. Due to the large sample size of the national survey and its very high weight, we did not enter its data into the analysis to estimate the overall combined mean current prevalence of MDD. Based on the results of 23 studies, the overall estimation for current prevalence of MDD was 4.1% (95% CI: 3.1-5.1). The findings showed substantial heterogeneity (τ^2^=96.3, df=22, p<0.001). The forest plot is shown in Figure 2. National information on current prevalence of MDD is limited to only one population based study that 250 clinical psychologists used SADS diagnostic tool for interviews. The study found out a prevalence of 1.8% for MDD.^22^

**Figure 3:**
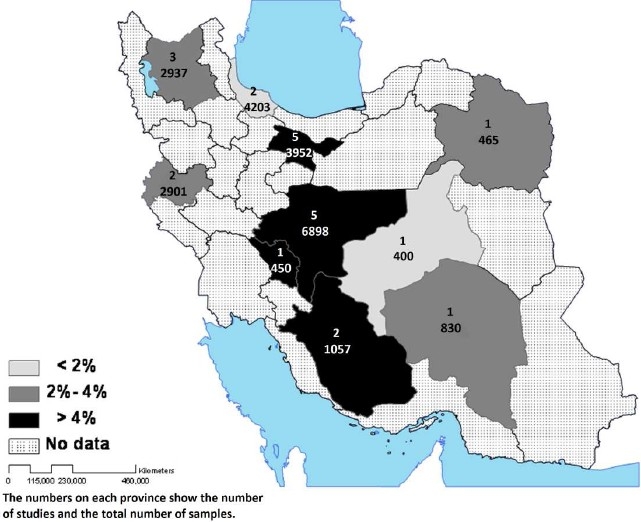
Spatial distribution of current prevalence of major depressive disorder in Iran

The maximum current prevalence of MDD was in urban residents of Shahreza city in Isfahan province (13.6%), which used Beck questionnaire and DSM-III based clinical interview; while the minimum reported prevalence of MDD was 0.25% in rural population of Meibod city in Yazd using SCL-90 questionnaire and DSM-III-R based clinical interview. The data has been available for 10 provinces. The combined mean prevalence for MDD was calculated for each province. Spatial distribution of the results is shown in Figure 3.

The meta-regression analysis showed that study date and publication date of the included studies as well as the living area of sample population (urban/rural) did not explain the variation between the findings of studies significantly. The analysis identified the variations in diagnostic tools as an important source of heterogeneity.

### Stratified pooled prevalence

**Figure 4:**
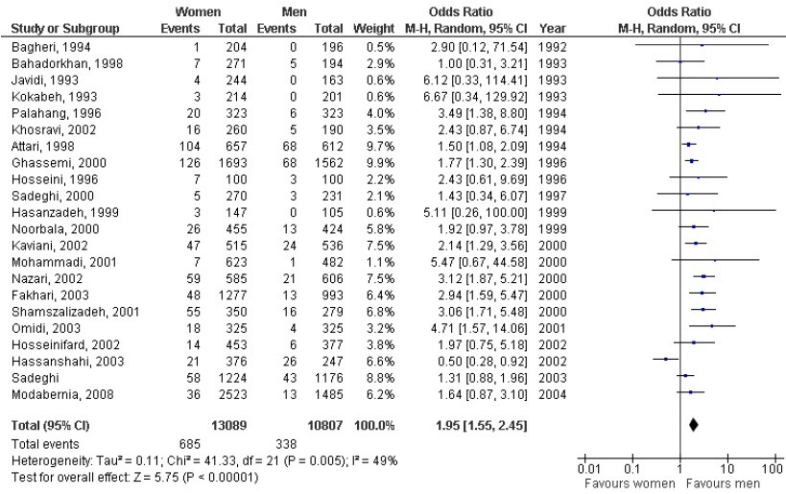
Risk of major depressive disorder among women compared with men in Iran

We stratified the included studies based on gender, the living area and the utilized tools and the results are presented below. Twenty-two studies have reported the current prevalence of MDD in both genders. Generally, the combined mean prevalence of MDD was greater in women (5.1%, 95% CI: 3.8-6.4) than in men (2.53%, 95% CI: 1.7-3.3). Figure 4 shows the odds ratio of MDD for women versus men. There was significant statistical heterogeneity (*χ*^2^=41.33, P=0.005). The meta-analysis showed that women were 1.95 (95% CI: 1.55-2.45) times more likely to have MDD.

**Figure 5:**
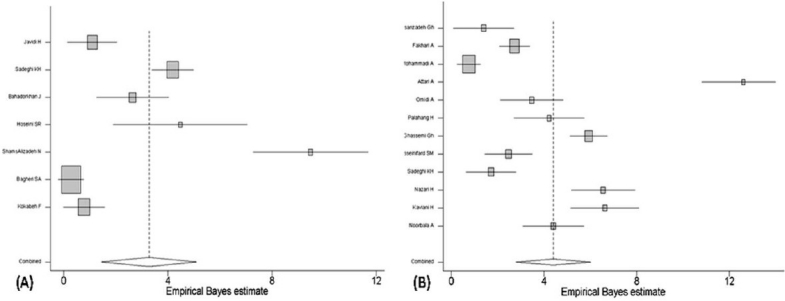
The current prevalence of major depressive disorder in rural (A) and urban (B) areas in studies conducted in Iran

Out of 23 studies, the sample population of 12 studies lived in urban areas and seven studies lived in rural areas. The living area was mixed in other three studies. The findings of both categories had considerable heterogeneity (τ^2^=95.0, df=11 and 95.7, df=6, respectively with p<0.001). The estimated prevalence of MDD, using random effect model was 3.3% (95% CI: 1.4-5.1) and 3.6% (95% CI: 2.3-4.9) in rural and urban samples, respectively. The forest plots are shown in Figures 5A and 5B. However, in the national survey, the current prevalence of MDD was reported to be significantly higher in rural areas than in urban areas (odds ratio=1.33, 95% CI: 1.10-1.61).

Five main diagnostic tools were used in the included studies; hence, we decided to estimate the tool-specific prevalence of MDD. The subgroup analysis revealed that reported current prevalence for MDD was higher when the utilized screening tool was Beck or GHQ-28 (Table 2).

**Table 2:** The tool-specific current prevalence of major depressive disorder

Tool	No. of studies	Prevalence	95% CI
SCL	9	2.01	1.08 – 2.94
Duke	1	2.7	2.03 – 3.37
SRQ	3	3.95	1.66 – 6.24
GHQ	6	5.62	3.72 – 7.52
Beck	4	7.03	1.75 – 12.31

## DISCUSSION

Prevalence rate is one of the most useful information for planning secondary and tertiary prevention activities^46^ along with studies on burden of diseases. This study was the first and the only systematic review addressing the prevalence of MDD in Iran. Although, publication bias is one of the main issues in most of the meta-analyses that may distort the findings, in this study we searched all available data sources to cover grey literature, as well. Our present systematic review and meta-analysis revealed a current prevalence of 4.1% for MDD in Iran, which is in a medium range comparing with other countries. It is higher than that in countries like china (2%)^14^ and Japan (2.9%)^15^ and lower than that in USA (6.6%)^13^ and Ukraine(8.3%).^8^ The result of the meta-analysis (4.1%) is much higher than the result of the national study (1.8%) on current prevalence of MDD. While the included studies in our meta-analysis had used a two step diagnostic procedure, in the national study SADS diagnostic tool was utilized in one step. The meta-analysis showed that the other studies were conducted in 10 out of 30 provinces. In general, the other 20 provinces with lack of data included provinces in borders of Iran and provinces that suffer from lower socioeconomic status and more deprivation. However, it is not clear if the difference between the results of national survey with the result of meta-analysis is because of the difference in methods and diagnostic tools, or the difference in sample population or the quality of the studies. The only evidence we found for lifetime prevalence of MDD was the rate of close to 3% reported in the national survey. As the result of the national survey for current prevalence of MDD was much lower than the combined mean prevalence of other studies, one can conclude that the rate of 3% for lifetime prevalence might be a low estimation as well. However, another study has also showed that recall bias can explain the unpredicted low level of lifetime prevalence of MDD.^47^ Heterogeneity in the findings of different studies could be due to many factors such as their sampling methods, the quality and the method of diagnosis or their different definitions of the disease in population, and their diversities in the age and gender.^48^,^49^ The differences might be due to the real variation in the prevalence of MDD in different provinces or the population. However, the use of different definitions (diagnostic tools) was the only source of found heterogeneity in our meta-analysis, although there might be other sources of heterogeneity, as well. A difference in the prevalence of MDD in women and men population was revealed in most included studies. The meta-analysis also showed that MDD was twice more prevalent in women than in men. This is consistent with the findings of the Iranian national survey on gender difference in lifetime prevalence of MDD. It is also consistent with evidence from the NCS-R and NCS^13^,^50^,^51^ and the other existing literature, which suggest about twice as many women as men experience an episode of MDD. In psychiatric epidemiology, higher prevalence of depression in women than in men is extensively documented and has been reported in studies using various diagnostic tools and interview methods.^52^,^53^ Studies on depression in adolescents reported that this gender difference first time appears at about 11 to 14 years of age.^54^ The contributed factors explaining the gender difference of MDD prevalence might be biological, psychological and social.^53^ The most commonly studied factors were the differences in sex hormones, social and family roles, as well as psychological characteristics.^53^–^57^ Urbanization and the economic growth of Iran in recent decades and the evolution in the roles of Iranian women could have affected the prevalence of MDD. The results of our study showed that the current prevalence of MDD in urban inhabitants is not significantly different from that in rural inhabitants. In Mohammadi et al. national survey, although the lifetime prevalence of MDD was greater in urban residents, but the current prevalence was greater in rural areas.^22^ However, no reason was mentioned for this observation in their published reports. Other studies across the world have found that MDD is more prevalent in urban population.^13^,^58^–^60^ Due to their exposure with various risk factors related to urbanization, urban residents are more likely to have depressive disorders. However, the various results reported in the national survey and the different finding of the meta-analysis make it difficult to conclude and it needs more investigations. It should be noted that it is more than two decades that mental health program in Iran is merged into the primary health care system. In year 2005, 80.6% of rural population was under coverage of this program and treatment services for mental disorders including MDD have been available for this population.^61^

### Limitations

We should acknowledge the limitations of using meta-analysis technique in descriptive observational studies. The main limitation is that the estimated prevalence was not adjusted based on the size of the target population. However, it seems that meta-analysis could be a feasible and efficient method to utilize the findings of all relevant studies appropriately. There were greater number of studies that had assessed mental disorders and included MDD, but their reported data for including the studies in this systematic review was insufficient. Although, contacting authors to find complementary data was extensively done, but a few eligible studies were excluded because additional information was not received.

## CONCLUSION

In conclusion, the prevalence of MDD in general population of Iran seems to be considerable. Moreover, considering higher prevalence of MDD in Iranian women, the health care system should plan for preventive and interventional programs, especially for women and for urban areas. According to the lack of studies in a large part of Iran, it is necessary to produce appropriate data. Due to the limitations of the only national study conducted in the country, it seems that another high quality national study can overcome the existing inadequacies.
